# Esketamine induces ferroptosis and suppresses immune evasion in lung adenocarcinoma cells via the hsa_circ_0022392/miR-548b-3p/MAP7 axis

**DOI:** 10.1186/s41065-026-00713-7

**Published:** 2026-07-31

**Authors:** Fei Chai, Yang Zhang, Xing Chen, XiaoYun Geng, XinJuan Kang, Hua Wan, XiaoBing Du

**Affiliations:** 1 Department of Anesthesiology, Affiliated Hospital of Hebei University, No. 212 Yuhua East Road, Lianchi District, Baoding City, Hebei Province 071000 China; 2 Department of Emergency Medicine, Dingzhou People’s Hospital, Baoding City, Hebei Province 073000 China; 3 Department of General Surgery, Baoding Fourth Central Hospital, Baoding City, Hebei Province 072750 China

**Keywords:** Lung adenocarcinoma, Esketamine, Ferroptosis, Immune evasion, hsa_circ_0022392/miR-548b-3p/MAP7 signaling axis

## Abstract

**Background:**

Although Esketamine, an N-methyl-D-aspartate (NMDA) receptor antagonist, is widely used for anesthesia and analgesia and has shown anticancer activity, its effects on lung adenocarcinoma remain unclear. Circular RNAs have been implicated in the development and progression of lung adenocarcinoma. Therefore, this study aims to elucidate whether esketamine can influence lung adenocarcinoma progression by interfering with the circular RNA hsa_circ_0022392.

**Methods:**

Expression levels of hsa_circ_0022392, miR-548b-3p, and microtubule-associated protein 7 (MAP7), as well as transfection efficiency, were assessed by quantitative real-time polymerase chain reaction (qRT-PCR) and Western blot analysis. Cell viability was assessed using the Cell Counting Kit-8 assay. 5-ethynyl-2’-deoxyuridine (EdU) assay was employed to examine cell proliferation. Transwell assays were employed for analyzing cell migration and invasion capabilities. Intracellular Fe²⁺ levels, malondialdehyde (MDA), glutathione (GSH), and reactive oxygen species (ROS) were measured using commercial kits. Lactate dehydrogenase (LDH) release assays were adopted to evaluate CD8⁺ T-cell cytotoxicity. Cytokine levels were measured via ELISA. Bioinformatics analysis combined with RNA immunoprecipitation and dual-luciferase reporter assays were employed to confirm the target relationship between miR-548b-3p and hsa_circ_0022392 or MAP7.

**Results:**

Esketamine significantly inhibited proliferation, migration, and invasion (all *P* < 0.05), induced ferroptosis (*P* < 0.05), and suppressed immune evasion (*P* < 0.05). Notably, hsa_circ_0022392 expression was upregulated in lung adenocarcinoma tissues and cell lines (*P* < 0.01), and esketamine downregulated its levels (*P* < 0.05). Overexpressed hsa_circ_0022392 notably reversed esketamine’s inhibitory effect on lung adenocarcinoma cell progression (*P* < 0.05). Further studies revealed that hsa_circ_0022392 targeted and bound to miR-548b-3p, thereby upregulating MAP7 expression (*P* < 0.05). Interfering with this signaling axis effectively weakened the inhibitory effect of esketamine on lung adenocarcinoma progression (*P* < 0.05).

**Conclusion:**

In summary, esketamine promotes ferroptosis in lung adenocarcinoma cells through regulating the hsa_circ_0022392/miR-548b-3p/MAP7 axis and inhibits immune evasion, providing novel molecular targets and therapeutic strategies for the diagnosis and treatment of lung adenocarcinoma.

**Supplementary Information:**

The online version contains supplementary material available at https://doi.org/10.1186/s41065-026-00713-7.

## Introduction

According to the 2022 Global Cancer Statistics Report, lung cancer has the highest incidence and mortality rates, making it the leading cause of cancer-related deaths worldwide [1]. Lung adenocarcinoma represents the most prevalent histological subtype of lung cancer [2]. Notably, its incidence continues to rise annually. Despite the effectiveness of recent comprehensive clinical therapies in mitigating disease progression, overall patient survival rates remain low [3]. Therefore, identifying more promising molecular targets or signaling pathways is crucial for improving the current treatment landscape of lung adenocarcinoma.

Ketamine, an N-methyl-D-aspartate (NMDA) receptor antagonist, is widely used clinically for anesthesia, sedation, and cancer pain management. Its new derivative, esketamine, has been introduced into clinical practice due to its higher NMDA receptor binding affinity, potent analgesic efficacy, and better safety profile [4, 5]. Previous studies have shown that the NMDA receptor antagonist ketamine can inhibit proliferation and migration and induce apoptosis in various cancer types, including glioma, lung cancer, liver cancer, ovarian cancer, and pancreatic cancer [6–9]. Meanwhile, recent studies have suggested the anticancer potential of esketamine. Research has demonstrated that esketamine dose‑dependently inhibits proliferation, migration, and invasion and induces apoptosis in esophageal squamous cell carcinoma cells [10]. Wang et al. found that esketamine not only provides significant sedation and analgesia for perioperative lung cancer patients but also downregulates postoperative inflammatory cytokine levels in the tumor microenvironment, highlighting its potential intervention in lung cancer metastasis [11]. However, no study has yet clearly indicated whether it can effectively regulate lung adenocarcinoma progression. Therefore, this study aimed to investigate the effects of esketamine on lung adenocarcinoma cells.

CircRNAs, a type of non-coding RNA, are commonly found in eukaryotic cells, featuring a covalently closed circular structure [12]. As novel regulatory RNAs, circRNAs have been demonstrated to have a significant connection to the initiation and progression of different diseases, including neurological disorders, cardiovascular diseases, and cancers [13, 14]. Notably, circRNAs exhibit abnormal expression in multiple tumor types (gastric, breast, and colorectal cancers). Research indicates they can act as competitive endogenous RNAs (ceRNAs) by binding to miRNA response elements, functioning as miRNA sponges to regulate biological processes such as tumor cell proliferation, invasion, and migration [15–17]. This study found that hsa_circ_0022392 is elevated in lung adenocarcinoma, leading to the hypothesis that it likely regulates lung adenocarcinoma progression.

Microtubule‑associated proteins (MAPs) regulate cellular dynamics and stability by directly binding to microtubules and are essential for cell division, transport, signal transduction, and maintenance of cellular structure [18, 19]. Previous studies have indicated that altered expression of these proteins is closely associated with migration and invasion potential and chemotherapy sensitivity in various cancer cells. Microtubule-associated protein 7 (MAP7) is also considered a key oncogenic factor [20, 21]. Studies have shown that MAP7 drives malignant phenotypes such as growth and metastasis in breast, ovarian, and cervical cancer cells, and its upregulation is associated with poor prognosis in cancer patients [22–25]. Meanwhile, MAP7 has been confirmed to participate in the growth, invasion, and chemotherapy resistance of lung adenocarcinoma cells [26], and is significantly enriched in non‑small cell lung cancer tissues [27]. However, the upstream regulatory mechanism of MAP7 remains unclear.

In this study, esketamine significantly suppressed malignant biological behaviors in lung adenocarcinoma cells while promoting ferroptosis and inhibiting immune evasion. To further elucidate its mechanism, bioinformatics analysis, clinical samples, and molecular biology experiments were used to reveal that esketamine regulates hsa_circ_0022392 expression. This circRNA further binds to miR-548b-3p, thereby modulating the expression of its downstream target gene MAP7. This cascade ultimately achieves effective regulation of biological processes in lung adenocarcinoma cells.

## Methods

### Collection of lung adenocarcinoma tissue samples

This study obtained lung adenocarcinoma tissue samples (*n* = 30) and neighboring non-cancerous tissue samples (*n* = 30) from individuals undergoing surgical treatment at Affiliated Hospital of Hebei University. All specimens were immediately cryopreserved at -80 °C following rapid freezing for subsequent processing. The present study was approved by the Ethics Committee of Affiliated Hospital of Hebei University and written informed consent was provided by all patients prior to the study start. All procedures were performed in accordance with the ethical standards of the Institutional Review Board and The Declaration of Helsinki, and its later amendments or comparable ethical standards. Clinical patient information is presented in Table 1.

**Table 1:** Correlation between hsa_circ_0022392 expression and clinical characteristics in lung adenocarcinoma patients

Parameters	*N*	hsa_circ_0022392 expression		*P* value
		Low (*n* = 30)	High (*n* = 30)	
Age				0.196
< 60	29	12	17	
≥ 60	31	18	13	
Gender				0.432
Male	35	19	16	
Female	25	11	14	
Tumor size				0.003
<3 cm	39	25	14	
≥3 cm	21	5	16	
TNM stage				0.038
I/II	32	20	12	
III/IV	28	10	18	
Lymph node metastasis				0.001
Yes	34	10	24	
No	26	20	6	

### Cell culture and processing

Human lung adenocarcinoma cell lines A549, HCC827, H1299, and H1975 were obtained from Procell (Wuhan, China). HBE cells were from ATCC (Manassas, VA, USA). Cells were cultured in RPMI 1640 medium (Gibco, Grand Island, NY, USA) containing 10% fetal bovine serum (FBS; Excell Bio, Shanghai, China) and 1% penicillin-streptomycin (Beyotime, Shanghai, China), and maintained in a humidified incubator at 37 °C with 5% CO₂. To assess the role of ferroptosis, cells were pretreated with 2 µM ferrostatin-1 (Fer‑1; Medchemexpress, Shanghai, China) for 24 h.

Activated CD8⁺ T cells were prepared from peripheral blood mononuclear cells (PBMCs) isolated from venous blood of healthy human donors, using kits from STEMCELL Technologies (Vancouver, BC, Canada) following manufacturer’s instructions. Subsequently, the activated cells were incubated in RPMI 1640 medium containing 10% FBS and 1% penicillin-streptomycin for further experiments.

### Cell transfection

siRNAs targeting hsa_circ_0022392 or MAP7, overexpression plasmids (ov-hsa_circ_0022392, ov-MAP7), miR-548b-3p mimics and inhibitors, and their negative controls (si-NC, ov-NC, mimic-NC, inhibitor-NC) were purchased from Sangon Biotech (Shanghai, China). The plasmids or siRNAs were transfected into A549 cells using Lipofectamine 2000 (Invitrogen, Carlsbad, CA, USA) according to manufacturer’s instructions. Following twenty-four-hour transfection, transfection efficiency was assessed via quantitative real-time polymerase chain reaction (qRT-PCR) or Western blot assay, followed by subsequent experiments.

### Cell counting kit-8 (CCK-8)

Cell viability was detected via CCK-8 assay kits. Treated A549 or HCC827 cells were collected to seed into a 96-well plate with a density of 1 × 10³ cells per well. After exposure to different concentrations (0, 0.4, 0.6, 0.8, 1, 1.2 mM) of esketamine (dissolved in physiological saline, Jiangsu Hengrui Pharmaceutical Co., Ltd., China) [5], lung adenocarcinoma cells were cultured for 24–48 h. Then they received incubation at 37 °C for 30 min following addition of 100 µL of CCK-8 reagent (Biosharp, Guangzhou, China) to each well, and absorbance at 450 nm was measured by microplate readers.

### 5-ethynyl-2’-deoxyuridine (EdU) assay

Cell proliferation capacity was assessed using EdU detection kits (Beyotime). Processed A549 or HCC827 cells were collected and seeded into 6-well plates. After overnight incubation, EdU reagent was added for continued incubation at room temperature for 2 h. Subsequently, 4% paraformaldehyde solution was added for fixation at room temperature for 15 min prior to a 10-minute permeabilization using 0.3% Triton X-100, and a 30-minute incubation with Click reaction solution at room temperature in the dark. Finally, cell nuclei were dyed with DAPI reagent (Biosharp) for 5 min. After washing with PBS, fluorescence microscopes (Olympus, Tokyo, Japan) were used to observe and image the cells.

### Transwell assay

Cell invasion and migration capabilities were evaluated using Transwell assay. The lower chamber of a 24-well Transwell plate was filled with 600 µL of RPMI 1640 medium containing 10% FBS. Prepared A549 or HCC827 cells were suspended in 100 µL of serum-free medium and evenly placed into the upper chamber. For the invasion assay, the upper chamber surface was pre-coated with diluted Matrigel from BD Biosciences (Franklin Lakes, NJ, USA). After incubating the cells in the incubator for 24 h, invading cells were fixed with 4% paraformaldehyde and stained with 0.1% crystal violet. At last, images of invading cells were observed and captured under optical microscopes.

### Lactate dehydrogenase (LDH) cytotoxicity assay

Obtained CD8⁺ T cells with A549 or HCC827 cells at a 5:1 ratio were co-cultured in a medium with no serum at 37 °C for 24 h. Subsequently, LDH activity was measured at 490 nm wavelength using CyQUANT LDH Cytotoxicity Detection Kit (Thermo Fisher Scientific, Waltham, MA, USA).

### ELISA

Cytokines CXCL9, IFN-γ, TNF-α, and IL-2 levels in the co-culture medium of CD8⁺ T cells and lung adenocarcinoma cells were detected using ELISA kits (Abcam, Cambridge, UK). The experimental procedures strictly followed the manufacturer’s instruction manual, and the absorbance values were finally read at a wavelength of 450 nm.

### qRT-PCR

Total RNA from lung adenocarcinoma cells in different treatment groups was extracted using Trizol reagent (Thermo, USA) and converted into cDNA with reverse transcription kits. Subsequently, PCR detection was conducted using SYBR Green PCR Master Mix (Invitrogen) on an Applied Biosystems™ 7500 Fast Real-Time PCR System (Foster City, CA, USA). Expression levels of circular RNA and mRNA were normalized using GAPDH as an internal reference, while miRNA expression level normalization was conducted using U6 as an internal reference. Gene expression levels were calculated using 2^−ΔΔCt^ method. Primer sequences are shown in Table 2.

**Table 2:** Primer sequences

Gene	Forward primer (5′-3′)	Reverse primer (5′-3′)
hsa_circ_0022392	5′-CGGGACCCTTTGTTTGTGTG-3′	5′-GCGGTAGATGGGGCTCAAAT-3′
miR-548b-3p	5′-GCGCAAGAACCTCAGTTGC-3′	5′-AGTGCAGGGTCCGAGGTATT-3′
MAP7	5′-CTGCGTGTCTGTAGTAGGGC-3′	5′-GGCTTCTGGCTCCTTTCCAT-3′
GAPDH	5′-GAAATGAATGGGCAGCCGTT-3′	5′-GACTCCACGACGTACTCAGC-3′
U6	5′-CTCGCTTCGGCAGCACA-3′	5′-AACGCTTCACGAATTTGCGT-3′

### Western blot assay

Using RIPA lysis buffer (Beyotime), total proteins were extracted from lung adenocarcinoma cells. Protein concentration determination was done using BCA Protein Quantification Kit (Biosharp). Proteins were separated on a 10% SDS-PAGE gel and then transferred to PVDF membranes. After blocking with 5% skim milk for 2 h, the membranes were incubated with primary antibodies MAP7 (DF15797, Affinity, Cincinnati, OH, USA), GPX4 (30388-1-AP, Proteintech, Rosemont, IL, USA), or GAPDH (10494-1-AP, Proteintech) at 4 °C overnight, and secondary antibody (SA00001-2, Proteintech) at room temperature for 1 h. Finally, the results were visualized using enhanced ECL chemiluminescence detection kits (Epizyme, Shanghai, China).

### Detection of Malondialdehyde (MDA), Glutathione (GSH), Fe²⁺ and Reactive Oxygen Species (ROS)

Strictly following the kit instructions, processed lung adenocarcinoma cells were collected to measure intracellular levels of MDA, GSH, and Fe²⁺ using the MDA (Beyotime), GSH (Beyotime), and Fe²⁺ (Sigma-Aldrich, St. Louis, MO, USA) assay kits. For ROS detection, cells were incubated with DCFH-DA reagent (Beyotime) at 37 °C for 30 min before washing three times with serum-free medium. ROS measurement was performed using fluorescence microplate readers (Thermo Fisher Scientific).

### Dual-luciferase assay

Using the CircInteractome and starBase databases, binding sites between miR-548b-3p and hsa_circ_0022392 or MAP7 were predicted. The pmirGLO reporter vectors (Promega, Madison, WI, USA) containing WT or MUT hsa_circ_0022392 and MAP7 with miR-548b-3p binding sites were constructed. Lung adenocarcinoma cells were seeded into 96-well plates (6 × 10³ cells/well). After 24-hour incubation in cell culture incubators, the luciferase reporter plasmid with miR-548b-3p mimic or negative control was co-transfected into lung adenocarcinoma cells using Lipofectamine 2000 and cultured for 48 h. Luciferase activity was detected using dual-luciferase assay system.

### RIP assay

The RIP assay was performed using the Magna RIP RNA-Binding Protein Immunoprecipitation Kit (Millipore, Burlington, MA, USA). The A549 cells were lysed with RIP lysis buffer. Ten microliters of cell lysate with magnetic beads conjugated to anti-Ago2 antibody (ACCm, Boston, MA, USA) or anti-IgG (as negative control, ACCm) were incubated at 4 °C for 6 h. The immunoprecipitated material bound to the magnetic beads was eluted using elution buffer. The immunoprecipitation products were stored for subsequent qRT-PCR analysis.

### Statistical analysis

All experiments were independently repeated three times. Data analysis was performed using GraphPad Prism 8 software. All results are presented as mean ± standard deviation. Differences between groups were analyzed using *t*-tests or one-way analysis of variance (ANOVA) followed by Tukey’s post hoc test. Pearson’s chi-square test was performed to assess the association between hsa_circ_0022392 expression and clinicopathological features. Results are considered statistically significant at *P* < 0.05.

## Results

### Esketamine inhibits lung adenocarcinoma cell viability, proliferation, and invasion

**Fig. 1:**
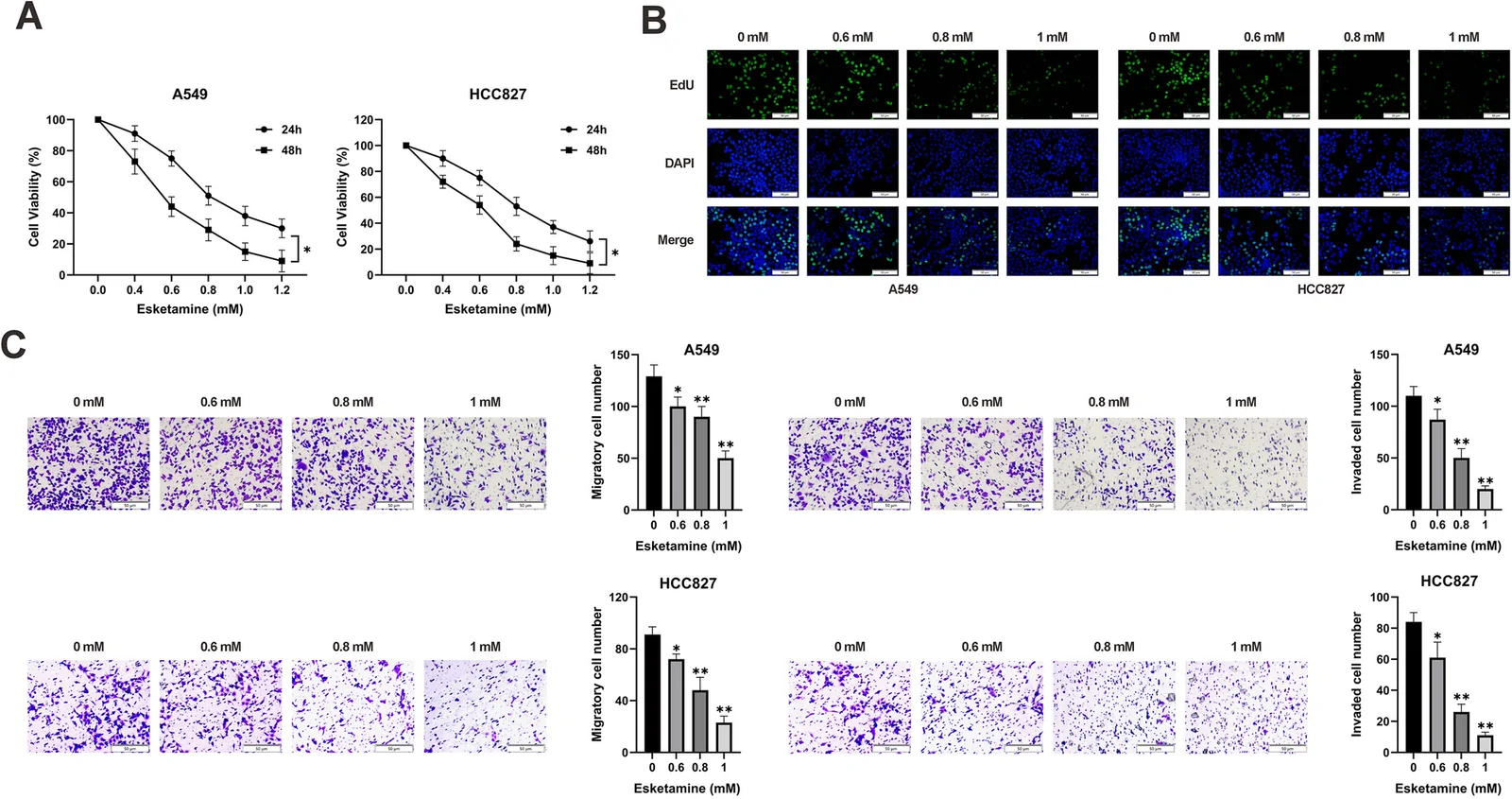
Esketamine inhibits lung adenocarcinoma cell viability, proliferation, and invasion. **A**: CCK-8 assay assessing the effects of esketamine at different concentrations (0, 0.4, 0.6, 0.8, 1, 1.2 mM) on A549 and HCC827 cell viability; **B**: EdU assay for cell proliferation; **C**: Transwell assay for A549 and HCC827 cell invasion, and migration capacity. Data are shown as mean ± standard deviation (n = 3). *P < 0.05, **P < 0.01 vs. 24 h or 0 mM esketamine group

This study first examined how esketamine affects lung adenocarcinoma cell viability. Esketamine dose-dependently reduced cell viability and EdU-positive cell numbers in A549 and HCC827 cells (Fig. 1A-B). Transwell assay results demonstrated that the numbers of invasive and migratory cells in the esketamine group were significantly lower than those in the control group (Fig. 1C). Therefore, esketamine dose-dependently inhibited the proliferation, invasion, and migration of lung adenocarcinoma cells.

### Esketamine induces ferroptosis and inhibits immune evasion in lung adenocarcinoma cells

**Fig. 2:**
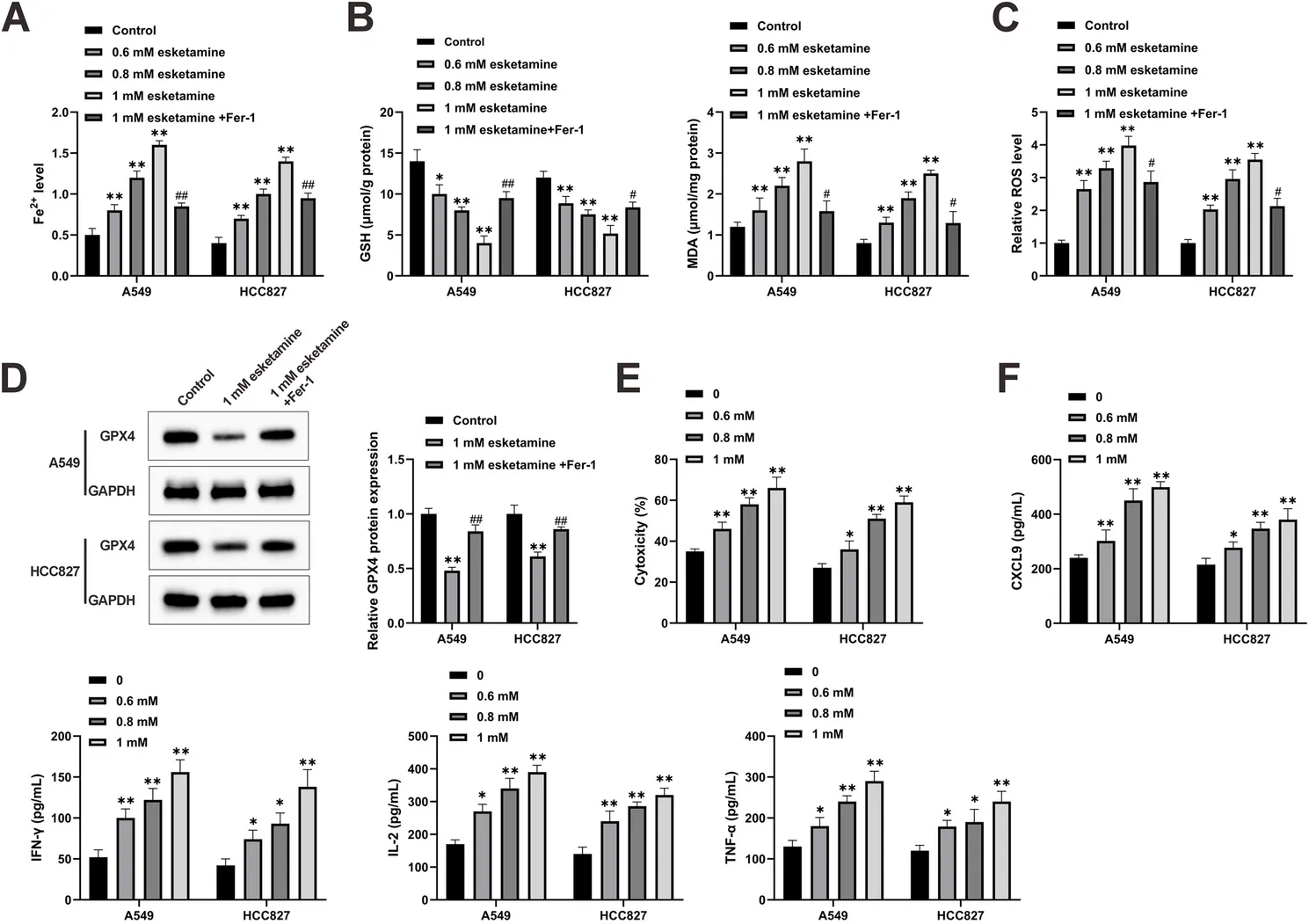
Esketamine Induces Ferroptosis and Inhibits Immune Evasion in Lung Adenocarcinoma Cells. **A**: Detection of Fe²⁺ levels in A549 and HCC827 cells; **B**: Kit-based detection of MDA and GSH levels in A549 and HCC827 cells; **C**: Kit-based detection of intracellular ROS levels; **D**: Western blot assay for GPX4 expression; **E**: LDH release assay for esketamine-treated CD8⁺ T cell cytotoxicity; **F**: ELISA for esketamine-treated CXCL9, IFN-γ, IL-2, and TNF-α levels. Data are shown as mean ± SD (*n* = 3). *P < 0.05, **P < 0.01 vs. Control group. #*P* < 0.05, ##*P* < 0.01 vs. 1 mM esketamine group

Previous studies have shown that ketamine can trigger ferroptosis in cancer cells. The ability of esketamine to induce ferroptosis in lung adenocarcinoma cells was evaluated. Compared with the control group (Fig. 2A-C), esketamine-treated A549 and HCC827 cells exhibited increased Fe²⁺, MDA, and ROS levels, decreased GSH levels, and downregulated GPX4 expression, a ferroptosis marker (Fig. 2D). However, the addition of the ferroptosis inhibitor Fer-1 reversed these changes. Previous studies have suggested that tumor cells may interact with CD8⁺ T cells in the microenvironment to suppress their cytotoxic function, thereby promoting malignant progression. Therefore, A549 and HCC827 cells were co‑cultured with CD8⁺ T cells, and LDH assays were performed to evaluate the anti-tumor immune effects of esketamine. The results demonstrated that esketamine significantly enhanced CD8⁺ T cell-mediated killing of lung adenocarcinoma cells (Fig. 2E). ELISA analysis further confirmed that, compared with the control group, esketamine treatment upregulated CXCL9, IFN-γ, IL-2, and TNF-α levels in the co-culture supernatant (Fig. 2F). These changes indicated enhanced CD8⁺ T cell activity. Thus, esketamine induced ferroptosis in lung adenocarcinoma cells and effectively suppressed tumor cell immune evasion.

### hsa_circ_0022392 is upregulated in lung adenocarcinoma tissues and cells

**Fig. 3:**
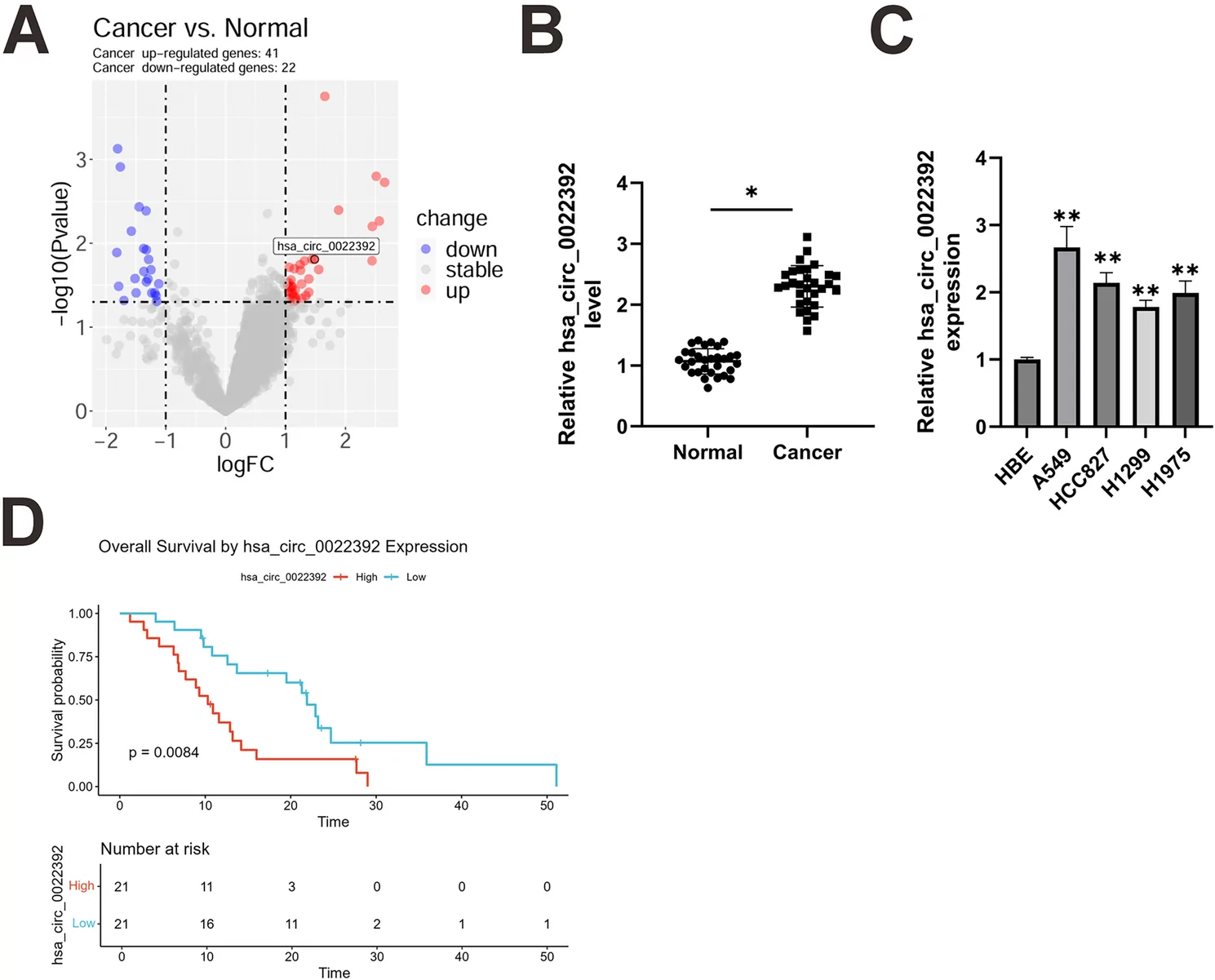
hsa_circ_0022392 Is Upregulated in Lung Adenocarcinoma Tissues and Cells. **A**: Expression profile of hsa_circ_0022392 from 5 lung adenocarcinoma tissue samples and 5 adjacent normal lung tissue samples in GSE101586 dataset; **B**: qRT-PCR detecting hsa_circ_0022392 expression levels in tumor tissue and normal lung tissue from lung adenocarcinoma patients (*n* = 30); **C**: qRT-PCR detecting hsa_circ_0022392 expression levels in lung adenocarcinoma cell lines and normal lung epithelial cells; **D**: Correlation analysis of survival rates in lung adenocarcinoma patients with high or low hsa_circ_0022392 expression. Data are presented as mean ± standard deviation. *P < 0.05, **P < 0.01 vs. Normal or HBE cell group

To identify potential targets of esketamine in lung adenocarcinoma cells, the GSE101586 dataset (comprising 5 lung adenocarcinoma tissue samples and 5 adjacent normal lung tissue samples) and patient tissue samples were used for analysis. Results revealed that hsa_circ_0022392 exhibited notably higher expression in lung adenocarcinoma tissues than in normal lung tissues (Fig. 3A-B). qRT-PCR results further confirmed that hsa_circ_0022392 was upregulated in different lung adenocarcinoma cell lines (A549, HCC827, H1299, and H1975), with the highest levels observed in A549 cells (Fig. 3C). Therefore, A549 cells were selected for subsequent experiments. Clinicopathological correlation showed that hsa_circ_0022392 expression was associated with tumor size, TNM stage, and lymph node metastasis (Table 1), and high expression correlated with poorer overall survival (Fig. 3D). These findings indicate that hsa_circ_0022392 is elevated in lung adenocarcinoma tissues and cells and is associated with poor prognosis.

### Esketamine Influences Lung Adenocarcinoma Progression by Regulating hsa_circ_0022392 Levels

**Fig. 4:**
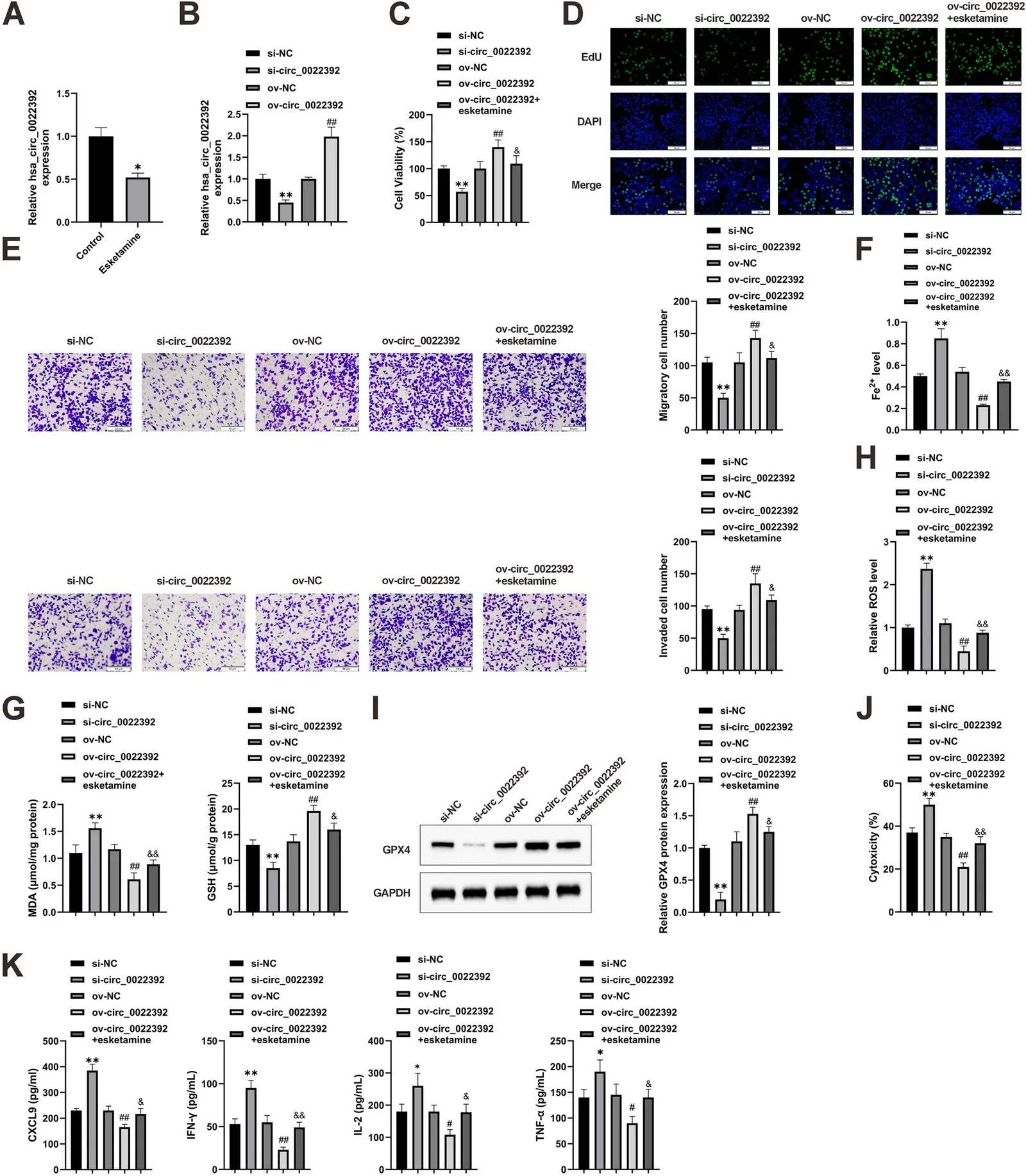
Esketamine Influences Lung Adenocarcinoma Progression by Regulating hsa_circ_0022392 Levels. **A**: qRT-PCR detection of hsa_circ_0022392 expression; **B**: qRT-PCR for verifying hsa_circ_0022392 transfection efficiency; **C**: CCK-8 assay for A549 cell viability; **D**: EdU assay for cell proliferation; **E**: Transwell assay for A549 cell invasion and migration; **F**: Kit assay for intracellular Fe²⁺ levels in A549 cells; **G**: Kit-based detecting MDA and GSH levels in A549 cells; **H**: Kit-based detection of intracellular ROS levels; **I**: Western blot analysis of GPX4 expression; **J**: LDH release assay to assess CD8⁺ T cell cytotoxicity after esketamine treatment; **K**: ELISA detection of CXCL9, IFN-γ, IL-2, and TNF-α levels. Data are shown as mean ± standard deviation (*n* = 3). *P < 0.05, **P < 0.01 vs. Control or si-NC group; #*P* < 0.05, ##*P* < 0.01 vs. ov-NC group; &*P* < 0.05, &&*P* < 0.01 vs. ov-hsa_circ_0022392 group

Compared with the control group, qRT-PCR results revealed that esketamine downregulated hsa_circ_0022392 expression in A549 cells (Fig. 4A). To further clarify how esketamine acts on hsa_circ_0022392, A549 cells were transfected with overexpression plasmids or siRNA targeting hsa_circ_0022392, and transfection efficiency was verified (Fig. 4B). CCK-8, EdU, and Transwell assays revealed that knockdown of hsa_circ_0022392 expression inhibited A549 cell viability, reduced the number of EdU-positive cells, and markedly decreased cell migration and invasion, whereas overexpression of hsa_circ_0022392 produced opposite effects. Notably, esketamine significantly attenuated the promotion of A549 cell proliferation, invasion, and migration by hsa_circ_0022392 overexpression (Fig. 4C-E). Additionally, ferroptosis and immune evasion levels were assessed in A549 cells under different treatment conditions. The results showed that, compared with si-NC, knockdown of hsa_circ_0022392 led to increased intracellular Fe²⁺, MDA, and ROS levels, decreased GSH levels, and downregulated GPX4 protein expression. Furthermore, co‑culture of transfected A549 cells with CD8⁺ T cells revealed that hsa_circ_0022392 knockdown enhanced CD8⁺ T-cell cytotoxicity and upregulated cytokine levels, as determined by LDH release assays, whereas hsa_circ_0022392 overexpression reversed these changes. Further treatment with esketamine reversed these changes (Fig. 4F-K). These findings affirm the significant impact of hsa_circ_0022392 on the malignant progression of lung adenocarcinoma cells and suggest that esketamine may modulate lung adenocarcinoma progression by interfering with hsa_circ_0022392.

### hsa_circ_0022392 targets and inhibits miR-548b-3p

**Fig. 5:**
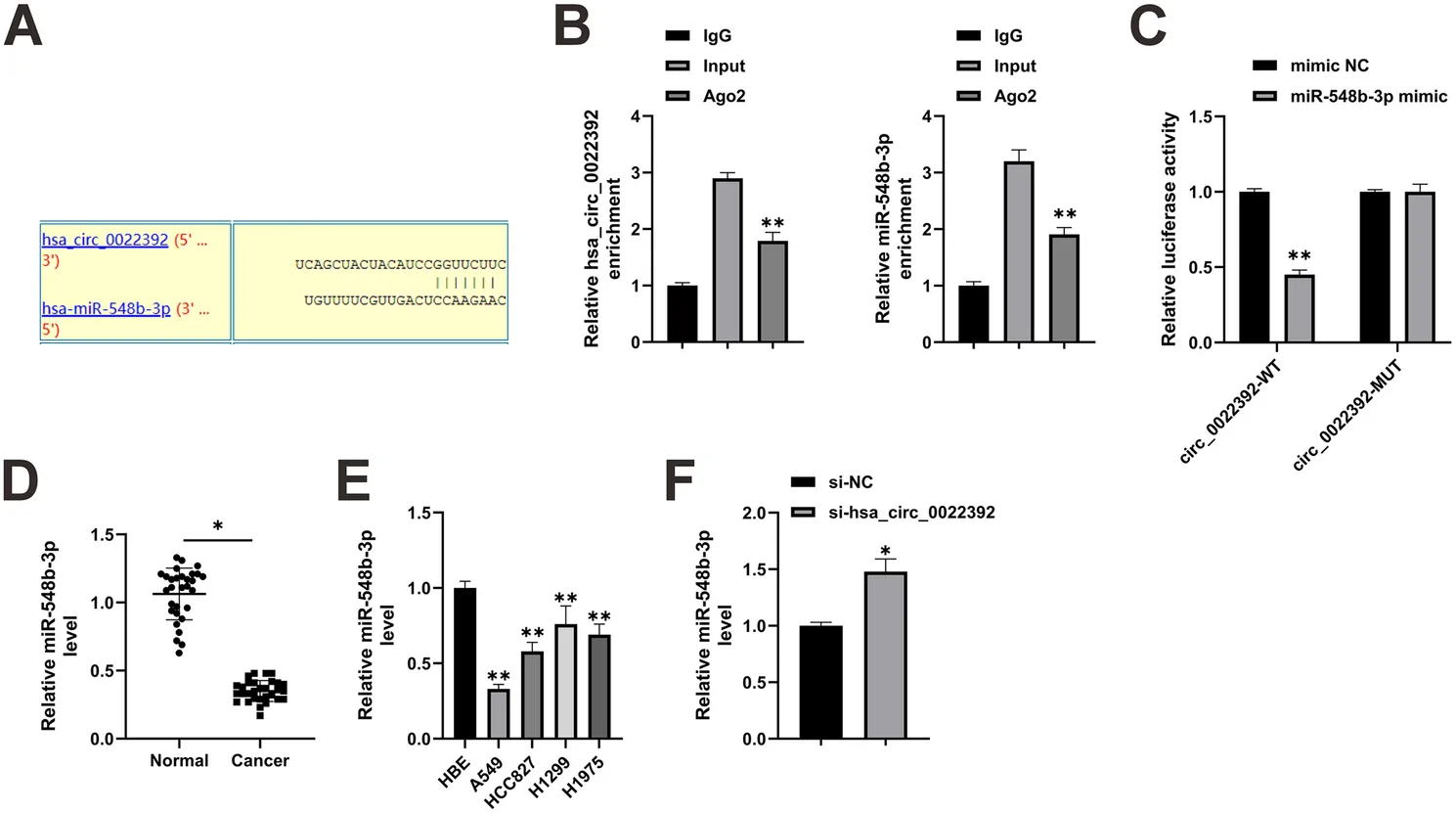
hsa_circ_0022392 Targets and Inhibits miR-548b-3p. **A**: CircInteractome website predicting binding sites between hsa_circ_0022392 and miR-548b-3p; **B**: RIP assay detects Ago2 enrichment of hsa_circ_0022392 and miR-548b-3p in A549 cells; **C**: Dual luciferase reporter assay verifying the target relationship between hsa_circ_0022392 and miR-548b-3p; **D**: qRT-PCR detection of miR-548b-3p levels in lung adenocarcinoma tissues (*n* = 30); **E**: qRT-PCR detection of miR-548b-3p levels in lung epithelial cells and lung adenocarcinoma cell lines; **F**: qRT-PCR detection of miR-548b-3p expression levels. Data are shown as mean ± standard deviation. *P < 0.05, **P < 0.01 vs. IgG, mimic NC, Normal, HBE cells, or si-NC group

To explore potential downstream targets of hsa_circ_0022392, CircInteractome (https://circinteractome.nia.nih.gov/) prediction revealed a binding site between hsa_circ_0022392 and miR-548b-3p (Fig. 5A). RIP assay results showed that both hsa_circ_0022392 and miR-548b-3p were highly enriched in the Ago2 fraction (Fig. 5B). Dual luciferase reporter assay results indicated that co-transfecting miR-548b-3p mimic and hsa_circ_0022392-WT considerably reduced luciferase activity, whereas no significant change was observed upon co-transfection with miR-548b-3p mimic and hsa_circ_0022392-MUT (Fig. 5C). Furthermore, this study found that miR-548b-3p levels were significantly lower in lung adenocarcinoma tissues compared to normal tissues, and its expression in lung adenocarcinoma cells was reduced relative to normal lung epithelial cells (Fig. 5D-E). Concurrently, qRT-PCR analysis revealed that knocking down hsa_circ_0022392 enhanced miR-548b-3p expression in A549 cells (Fig. 5F). These data indicate that hsa_circ_0022392 targets and suppresses miR-548b-3p.

### MAP7 Is a downstream target gene of miR-548b-3p

**Fig. 6:**
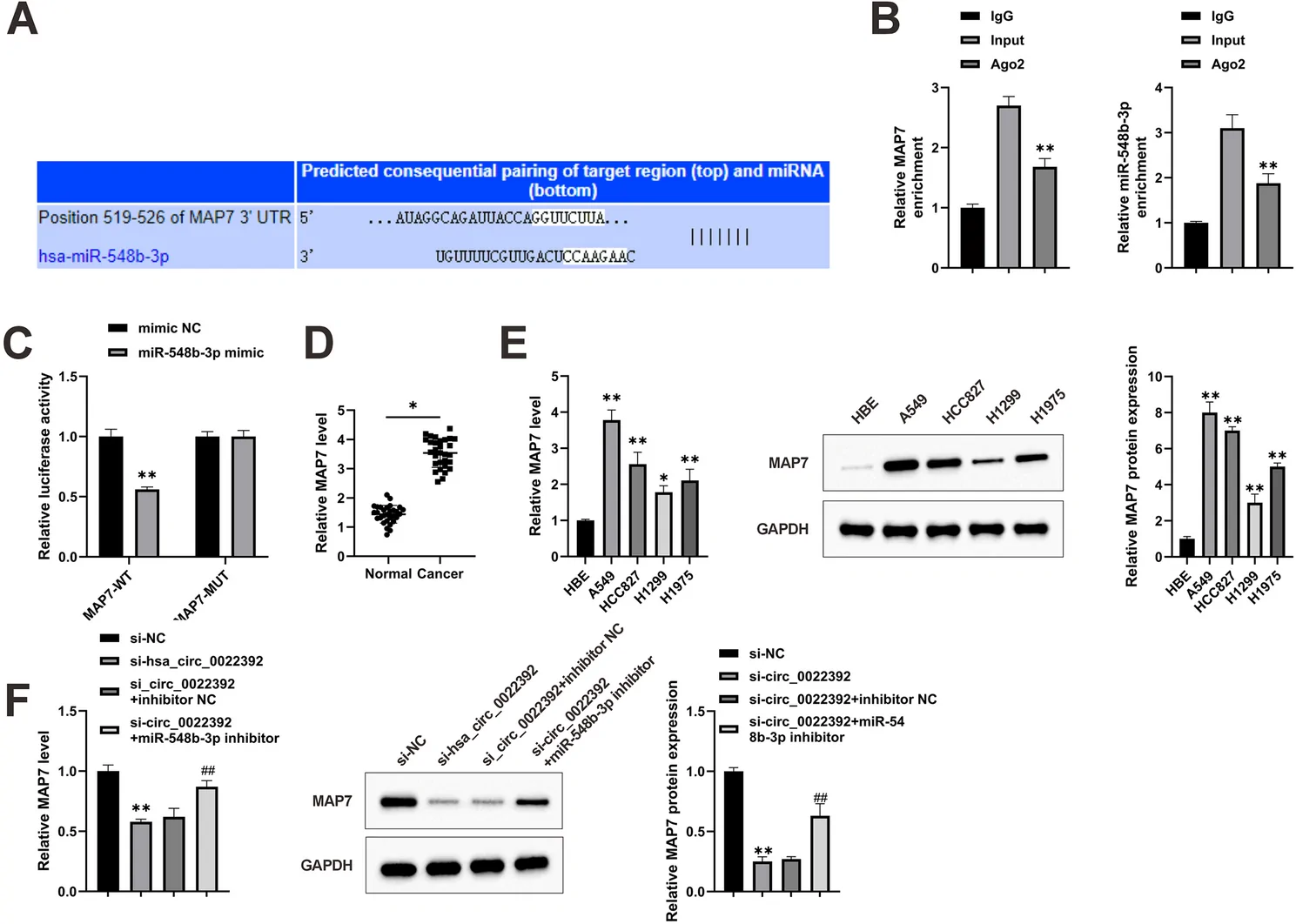
MAP7 Is a Downstream Target Gene of miR-548b-3p. **A**: starBase website predicting binding sites between miR-548b-3p and MAP7; **B**: RIP assay detecting miR-548b-3p and MAP7 enrichment by Ago2 in A549 cells; **C**: Dual-luciferase reporter assay validating miR-548b-3p and MAP7 binding; **D**: qRT-PCR detecting MAP7 levels in tumor versus normal tissues (*n* = 30); **E**: qRT-PCR and Western blot assessing MAP7 expression in lung adenocarcinoma cell lines and normal lung epithelial cells; **F**: MAP7 expression levels were detected by qRT-PCR and Western blot. Data are presented as mean ± standard deviation. *P < 0.05, **P < 0.01 vs. IgG, mimic NC, Normal, HBE cells, or si-NC group. ##*P* < 0.01 vs. si-hsa_circ_0022392 + inhibitor NC group

Prediction using starBase (https://rnasysu.com/encori/) revealed a potential binding site between microtubule-associated protein 7 (MAP7) and miR-548b-3p (Fig. 6A). RIP assay results showed significant enrichment of miR-548b-3p and MAP7 in Ago2 magnetic beads (Fig. 6B). Furthermore, dual luciferase reporter assays demonstrated markedly reduced luciferase activity in the miR-548b-3p mimic and MAP7-WT co-transfection group, whereas no notable change was seen in miR-548b-3p mimic and MAP7-MUT co-transfection groups (Fig. 6C). Moreover, we observed significant MAP7 overexpression in both lung adenocarcinoma tissues and cell lines (Fig. 6D-E). To determine the regulatory relationship among hsa_circ_0022392, miR-548b-3p, and MAP7, we first knocked down hsa_circ_0022392 levels in A549 and HCC827 cells, observing downregulated MAP7 levels. This suppression was partially reversed by adding miR-548b-3p inhibitor (Fig. 6F, Supplementary Fig. 1A). These results indicate that hsa_circ_0022392 acts as a ceRNA by sponging miR-548b-3p, thereby upregulating MAP7 expression.

#### The hsa_circ_0022392/miR-548b-3p/MAP7 axis mediates esketamine-induced ferroptosis and immune evasion in lung adenocarcinoma

**Fig. 7:**
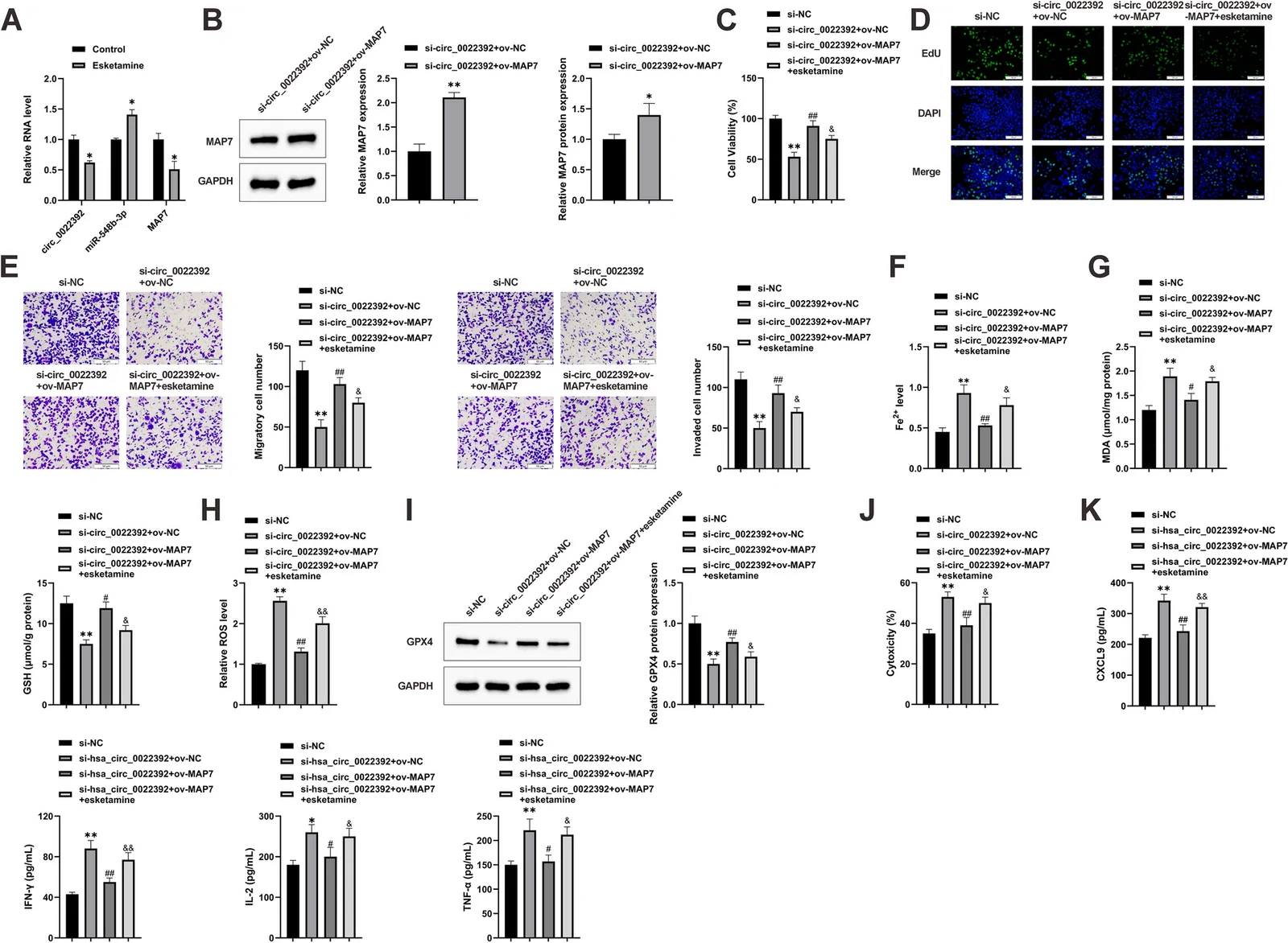
The hsa_circ_0022392/miR-548b-3p/MAP7 Axis Mediates Esketamine-Induced Ferroptosis and Immune Evasion in Lung Adenocarcinoma. **A**: qRT-PCR detecting hsa_circ_0022392, miR-548b-3p, and MAP7 levels in A549 cells; **B**: qRT-PCR and Western blot detecting MAP7 expression levels after co-transfection with si-hsa_circ_0022392 and ov-MAP7; **C**: CCK-8 assay for A549 cell viability; **D**: EdU assay for cell proliferation; **E**: Transwell assay for A549 cell invasion and migration; **F**: Fe²⁺ level detection in A549 cells; **G**: MDA and GSH level detection in A549 cells; **H**: Kit-based measurement of intracellular ROS levels; **I**: Western blot analysis of GPX4 expression; **J**: LDH release assay to assess esketamine-treated CD8⁺ T cell cytotoxicity; **K**: ELISA detecting CXCL9, IFN-γ, IL-2, and TNF-α levels. Data are shown as mean ± standard deviation (*n* = 3). *P < 0.05, **P < 0.01 vs. Control or si-NC group. #*P* < 0.05, ##*P* < 0.01 vs. si-hsa_circ_0022392 + ov-NC group. &*P* < 0.05, &&*P* < 0.01 vs. si-hsa_circ_0022392 + ov-MAP7 group

Compared with the control group, esketamine markedly downregulated MAP7 expression in A549 cells and upregulated miR-548b-3p expression (Fig. 7A). Given previous findings, it was hypothesized that esketamine may regulate lung adenocarcinoma progression via the hsa_circ_0022392/miR-548b-3p/MAP7 axis. Therefore, rescue experiments were conducted to verify this hypothesis. si-hsa_circ_0022392 and ov-MAP7 were co-transfected into A549 cells, and MAP7 expression levels were detected by qRT-PCR and Western blot assay (Fig. 7B). CCK-8, EdU, and Transwell assays revealed that downregulating hsa_circ_0022392 significantly inhibited A549 cell viability, suppressed proliferation, and reduced invasive and migratory cell numbers. Partial recovery of these parameters was observed upon MAP7 overexpression. However, esketamine treatment again inhibited A549 cell activity and function (Fig. 7C-E). Subsequent assessment of ferroptosis and immune evasion in A549 cells revealed that MAP7 overexpression attenuated the pro-ferroptotic effects of hsa_circ_0022392 silencing and partially reversed its suppression of immune evasion. Esketamine administration again reversed these effects (Fig. 7F-K). Rescue experiments were also performed in HCC827 cells, and the results were consistent with those in A549 cells (Supplementary Fig. 1A–L), confirming that esketamine mediates the hsa_circ_0022392/miR-548b-3p/MAP7 axis to inhibit lung adenocarcinoma cell proliferation, invasion, and migration, promote ferroptosis, and suppress tumor immune evasion.

## Discussion

Lung adenocarcinoma remains a major global health challenge and a leading cause of cancer-related mortality [28]. Existing therapeutic agents often cause adverse effects; therefore, identifying more effective and safer therapeutic targets and developing novel treatment strategies are crucial to improving clinical outcomes for patients [29–31]. This study demonstrates that esketamine significantly inhibits the proliferation, invasion, and migration of lung adenocarcinoma cell lines in vitro, while triggering ferroptosis and suppressing tumor immune evasion. These findings suggest that the anticancer potential of esketamine may be associated with targeting the intracellular circular RNA hsa_circ_0022392.

Esketamine is the S-enantiomer of the NMDA antagonist ketamine, exhibiting significant clinical advantages in the treatment of depression and cancer pain [32, 33]. Numerous studies have reported the significant anticancer efficacy of ketamine both in vitro and in vivo. Meanwhile, the potential of esketamine to interfere with malignant progression of cancer cells has been suggested. Li et al. demonstrated that esketamine inhibits proliferation, induces apoptosis, and suppresses invasion and migration in esophageal squamous cells [10]. However, direct evidence regarding the effects of esketamine on malignant phenotypes of lung adenocarcinoma is lacking. Notably, this study confirms that esketamine dose-dependently inhibits the malignant phenotypes of lung adenocarcinoma cells (A549/HCC827) and suppresses their migration and invasion capabilities. Thus, this study provides new evidence for the anticancer properties of esketamine.

circRNAs are single-stranded non-coding RNAs that are frequently dysregulated in cancers and act as key regulators of tumor progression [34]. These hold promise as biomarkers for cancer diagnosis or as targets for therapy [35]. For instance, circRNA hsa_circ_0003258 promotes prostate cancer metastasis in vivo and in vitro by binding to IGF2BP3 [36]. In esophageal cancer, circRNA CircBCAR3 accelerates tumorigenesis and metastasis by sponging miR-27a-3p [37]. In the present study, bioinformatics analysis revealed significantly elevated hsa_circ_0022392 expression in lung adenocarcinoma tissues and cell samples. Co-treatment with esketamine suppressed this circRNA expression level, while hsa_circ_0022392 overexpression partially counteracted the inhibitory effect of esketamine on malignant progression in lung adenocarcinoma cells. These findings suggest that esketamine may target this circRNA to regulate cancer biological behavior.

In recent cancer research, circRNAs typically function by sponging miRNAs, interacting with proteins, or being translated into proteins to regulate miRNA activity. The circRNA cMras suppresses the advancement of lung adenocarcinoma by regulating the miR-567/PTPRG pathway [38]. Additionally, hsa_circ_0000376 targets miR-1285-3p to participate in breast cancer development [39]. Therefore, to investigate the regulatory mechanism of hsa_circ_0022392, bioinformatics analysis platforms were utilized, and a binding site between hsa_circ_0022392 and miR-548b-3p was identified. Both RIP and dual luciferase reporter assays verified their binding. Furthermore, it was found that miR-548b-3p was abnormally downregulated in both lung adenocarcinoma tissues and cells. miR-548b-3p has been established as a tumor suppressor, regulating tumor proliferation, metastasis, apoptosis, and other processes in various cancers (lung, breast, colorectal, and liver cancers) [40–43]. These findings are consistent with previous reports on the role of miR‑548b‑3p in cancer progression. These findings confirm that hsa_circ_0022392 functions as a sponge for miR-548b-3p, suppressing its expression and thereby regulating downstream genes or signaling pathways.

To investigate the downstream target of hsa_circ_0022392 as a sponge for miR-548b-3p, MAP7 was identified as a likely target of miR‑548b‑3p through experimental validation. MAP7 belongs to the microtubule-associated protein family, participating in microtubule dynamics, regulating microtubule organization, and mediating microtubule interactions with other proteins [44]. Recent studies suggest that enhanced MAP7 expression accelerates migration and invasion in cervical, ovarian, and gastric cancer cells [23, 45, 46]. Consistent with this, elevated MAP7 levels are also observed in lung adenocarcinoma tissues and cells. As a ceRNA, hsa_circ_0022392 may bind to miR-548b-3p, thereby promoting an increase in MAP7 levels within A549 cells. Further rescue experiments revealed that MAP7 overexpression attenuated the inhibitory effects of hsa_circ_0022392 silencing on lung adenocarcinoma cell proliferation, migration, invasion, and immune evasion, as well as its promotion of ferroptosis. However, esketamine treatment again reversed these effects. Notably, the role of esketamine in ferroptosis remains controversial in the current literature. Previous studies have shown that esketamine exerts cytoprotective effects by inhibiting ferroptosis through anti‑oxidative stress in pulmonary epithelial cells with acute lung injury and in cardiomyocytes with ischemia‑reperfusion injury [47, 48]. However, the present study observed that esketamine significantly induced ferroptosis in the lung adenocarcinoma model. This discrepancy may be attributed to cell type specificity and the unique tumor microenvironment. In conclusion, esketamine, as a widely used NMDA receptor antagonist in clinical practice, has an extensive clinical safety record. The present study provides preclinical evidence for its therapeutic potential in lung adenocarcinoma. Given that esketamine effectively induces ferroptosis, future clinical trials could explore whether esketamine, as an adjuvant, synergistically induces ferroptosis when combined with kinase inhibitors like sorafenib or erlotinib.

Although the present study suggests the potential anticancer properties of esketamine, several limitations should be acknowledged. Future studies are required to verify the in vivo efficacy of esketamine using animal models. Additionally, since esketamine is a classic NMDA receptor antagonist, Lv et al. reported that GRIN2D, which encodes the NMDA receptor 2D subunit, is upregulated in lung adenocarcinoma cells and is involved in tumor cell proliferation and migration [49]. Therefore, the possibility that esketamine acts directly via NMDA receptor blockade and downstream signaling remains to be explored. Future investigations should include NMDA receptor agonists to further address this possibility.

## Conclusion

In summary, this study confirms the potential of esketamine to inhibit the biological behavior of lung adenocarcinoma cells, possibly through the hsa_circ_0022392/miR-548b-3p/MAP7 signaling pathway. These findings provide new mechanistic insights into the development of lung adenocarcinoma and offer potential therapeutic strategies for clinical anticancer treatment.

## Supplementary Information

Supplementary Material 1.

Supplementrary Material 2: Fig. s1 Esketamine Mediates the hsa_circ_0022392/miR‑548b‑3p/MAP7 Axis in Ferroptosis and Immune Evasion in HCC827 Cells. A: MAP7 expression levels were detected by Western blot. B: The levels of hsa_circ_0022392, miR‑548b‑3p, and MAP7 in HCC827 cells were measured by qRT‑PCR. C: MAP7 expression was detected by Western blot after co‑transfection with si‑hsa_circ_0022392 and ov‑MAP7. D: HCC827 cell viability was assessed by CCK‑8 assay. E: Cell proliferation was determined by EdU assay. F: Invasion and migration abilities of HCC827 cells were evaluated by Transwell assay. G: Intracellular Fe²⁺ levels in HCC827 cells were measured. H: MDA and GSH levels in HCC827 cells were detected. I: Intracellular ROS levels were examined using a kit. J: GPX4 expression was analyzed by Western blot. K: CD8⁺ T‑cell cytotoxicity after esketamine treatment was assessed by LDH release assay. L: Levels of CXCL9, IFN‑γ, IL‑2, and TNF‑α were measured by ELISA. Data are presented as mean ± SD (n = 3). *P < 0.05, **P < 0.01 vs. Control or si‑NC group; #P < 0.05, ##P < 0.01 vs. si‑circ_0022392 + ov‑NC group; &P < 0.05, &&P < 0.01 vs. si‑circ_0022392 + ov‑MAP7 group.

## Data Availability

The datasets used and/or analyzed during the present study are available from the corresponding author on reasonable request.
